# Circulating cell-free DNA level predicts all-cause mortality independent of other predictors in the Health 2000 survey

**DOI:** 10.1038/s41598-020-70526-9

**Published:** 2020-08-14

**Authors:** L. Kananen, M. Hurme, M. Jylhä, T. Härkänen, S. Koskinen, S. Stenholm, M. Kähönen, T. Lehtimäki, O. Ukkola, J. Jylhävä

**Affiliations:** 1 Faculty of Medicine and Health Technology (MET), Tampere University, Arvo Ylpön katu 34, Tampere, Finland; 2 Gerontology Research Center (GEREC), Tampere, Finland; 3 Faculty of Social Sciences (Health Sciences), Tampere University, Tampere, Finland; 4 Finnish Institute for Health and Welfare, Helsinki, Finland; 5 Department of Public Health, University of Turku and Turku University Hospital, Turku, Finland; 6 Centre for Population Health Research, University of Turku and Turku University Hospital, Turku, Finland; 7 Faculty of Medicine and Health Technology, Tampere University, and Finnish Cardiovascular Research Center, Tampere, Finland; 8 Department of Clinical Physiology, Tampere University Hospital, Tampere, Finland; 9 Department of Clinical Chemistry, Fimlab Laboratories, Tampere, Finland; 10 Research Unit of Internal Medicine, Medical Research Center Oulu, Oulu University Hospital, University of Oulu, Oulu, Finland; 11 Department of Medical Epidemiology and Biostatistics, Karolinska Institutet, Stockholm, Sweden

**Keywords:** Molecular biology, Medical research, Biomarkers, Risk factors

## Abstract

Increased levels of circulating cell-free DNA (cf-DNA) are associated with and predict poor health outcomes. However, its predictive ability for mortality in population-based samples remains understudied. We analysed the capability of cf-DNA to predict all-cause mortality and assessed whether it adds predictive value on top of the other risk factors in the Health 2000 survey (n = 1,257, 46–76 years of age, 15-years-follow-up, 18% deceased). When analysed in a multivariate model with the other factors that independently predicted mortality in the sample (age, gender, self-rated health, smoking and plasma levels of glucose and adiponectin), increases in cf-DNA levels were associated with increased risk of mortality (hazard ratio [HR] for 0.1 µg increase in cf-DNA: 1.017, 95% confidence interval [CI] 1.008–1.026, *p* = 0.0003). Inclusion of cf-DNA in the model improved the model fit and discrimination. Stratifying the analysis by cardiovascular disease (CVD) status indicated that cf-DNA predicted mortality equally well in individuals with (HR 1.018, 95% CI 1.008–1.026, *p* = 0.002) and without (HR 1.018, 95% CI 1.001–1.035, *p* = 0.033) CVD. In conclusion, our study indicates that cf-DNA level predicts mortality in middle-aged and older individuals, also among those with established CVD, and adds significant value to mortality prediction. Our results thus underscore the role of cf-DNA as a viable marker of health.

## Introduction

Circulating cell-free DNA (cf-DNA) level has been established as a sensitive, novel marker of cellular death and tissue damage in a variety of acute and chronic pathologies, such as sepsis, trauma, aseptic inflammation, cardiovascular diseases (CVDs) and cancer^1–6^. All of these conditions are characterised by elevated levels of cf-DNA in the blood, and higher cf-DNA level also predicts poorer outcomes in these conditions, independent of other risk factors. cf-DNA assessment may thus offer a new, minimally invasive prognostic tool. In cardiovascular medicine, measurement of cf-DNA level has already been put forward as an alternative method to monitor disease course and associated risk^7–9^. We have previously found that elevated cf-DNA level associates with several cardiometabolic risk factors, such as high blood pressure, unfavorable lipid profile and systemic inflammation^10^, suggesting that cf-DNA may also have utility in subclinical assessment of CVD risk.

Despite intensive research in recent years, the origins and mechanisms of tissue injury that lead to elevated cf-DNA level are only partially understood. Evidence suggests that both apoptosis and necrosis contribute to circulating cf-DNA, and even viable cells can under certain circumstances release DNA into circulation^11–14^. The biological properties of cf-DNA are likewise incompletely understood. There is, however, ample experimental evidence that extracellular DNA can act as a danger signal and stimulate immune responses. For example, in cardiopulmonary bypass patients, cf-DNA acts as an initiator of neutrophil activation and subsequent endothelial cell damage through triggering of NETosis. NETosis is a unique type of cell death whereby activated neutrophils release their extracellular traps, consisting DNA and histones, to the circulation^15^. However, whether the prognostic biomarker role of cf-DNA is coupled with, or due to, its potential damage-inducing effects is unknown.

In keeping with the observation that cf-DNA level is a broad biomarker of human health, we have previously found that it predicts 4-years all-cause mortality in very old individuals^16^. Characterisation of cf-DNA as a marker of general health status is nevertheless still in its infancy. Specifically, for its use in risk stratification, the capacity of cf-DNA level to predict mortality needs to be investigated at large in younger adult populations. In this study, we used a sample of the Finnish population-based Health 2000 survey that has an extensive array of health indicators (anthropometrics, conventional biomarkers, health behaviors and disease diagnoses) and mortality follow-up for 15 years available. From a set of 30 pre-defined variables that associate with health and/or mortality—including cf-DNA—we first identified those that, independently of each other, predict all-cause mortality. We also investigated whether cf-DNA level holds equal predictive potential in individuals with and without established CVD and assessed the added predictive value of cf-DNA.

## Results

The associations between cf-DNA, the other study variables (in Table 1) and all-cause mortality were analysed in a sample of 1,257 participants in the Health 2000 survey^17^. Causes of death are shown in Supplementary Table S1. The participants were followed-up to all-cause mortality for 15 years; 228 (18%) individuals died during that time. In the deceased individuals, the average survival time was 10.3 (standard deviation 4.2) years. Distributions of the study variables are shown in Table 1. Statistically significant differences between survivors and non-survivors were found in 19/30 study variables, including cf-DNA (Table 1).

**Table 1:** Participant characteristics.

	All	Survivors	Deceased	*p*
N (%)	1,257 (100)	1,029 (82)	228 (18)	
Age range (median)	46–76 (56)	46–76 (55)	47–76 (67)	< 0.001^M^
Female, N (%)	682 (54)	595 (58)	87 (38)	< 0.001^P^
Cell-free DNA, µg/ml*	0.84 (0.10)	0.84 (0.10)	0.87 (0.11)	< 0.001^M^
Adiponectin, µg/ml*	10 (5.1)	9.9 (5.0)	10.4 (5.4)	0.28^M^
Apolipoprotein A1, g/L*	1.7 (0.3)	1.8 (0.3)	1.7 (0.3)	0.0045^M^
Apolipoprotein B, g/L*	1.2 (0.2)	1.2 (0.3)	1.2 (0.2)	0.91^M^
CRP, mg/L*	1.5 (1.4)	1.5 (1.3)	1.8 (1.6)	0.0026^M^
Fasting glucose, mmol/L*	5.6 (0.6)	5.6 (0.6)	5.8 (0.6)	< 0.001^M^
Ghrelin, pg/ml*	6,200 (1,800)	6,300 (1,700)	5,900 (1,700)	< 0.001^M^
HDL cholesterol, mmol/L*	1.5 (0.4)	1.6 (0.4)	1.5 (0.4)	0.026^M^
IL-6, ng/L*	1.5 (0.9)	1.4 (0.7)	2.2 (1.5)	< 0.001^M^
Insulin, mU/L*	7.8 (3.7)	7.6 (3.6)	9.1 (4.8)	< 0.001^M^
LDL cholesterol, mmol/L*	3.7 (0.9)	3.7 (0.9)	3.6 (0.9)	0.21^M^
Resistin, ng/ml*	63 (32)	62 (32)	69 (36)	0.93^M^
TNF-alpha, ng/L*	5.7 (1.8)	5.5 (1.6)	6.5 (2.1)	< 0.001^M^
Total cholesterol, mmol/L*	5.6 (0.9)	5.6 (0.9)	5.5 (1.0)	0.043^M^
Triglycerides, mmol/L*	1.2 (0.6)	1.2 (0.6)	1.2 (0.4)	0.22^M^
Education, level*				< 0.001^P^
The lowest	490 (39)	367 (36)	123 (54)	
Middle	391 (31)	333 (32)	58 (25)	
The highest	376 (30)	329 (32)	47 (21)	
Smoking, N (%)	241 (19)	173 (17)	68 (30)	< 0.001^P^
Intensive exercise > 10 min, number of days/week*	1 (1.5)	1 (1.5)	1 (1.5)	0.1^M^
Consuming fresh vegetables, N (%)				0.004^P^
Never	63 (5)	42 (4)	21 (9)	
1–2 days/week	128 (10)	100 (10)	28 (12)	
3–5 days/week	225 (18)	182 (18)	43 (19)	
6–7 days/week	841 (67)	705 (69)	136 (60)	
Alcohol consumption, g/week*	27 (40)	28 (41)	23 (34)	0.19^M^
BMI (kg/m^2^)*	27 (4)	26 (4)	27 (4)	0.96^M^
Self-rated health, N (%)				< 0.001^P^
Good	425 (34)	380 (37)	45 (20)	
Rather good	421 (34)	342 (33)	79 (35)	
Moderate	342 (27)	266 (26)	76 (33)	
Rather poor	57 (5)	38 (4)	19 (8)	
Poor	12 (1)	3 (0.3)	9 (4)	
History of respiratory disease, N (%)	274 (22)	199 (19)	75 (33)	< 0.001^P^
History of CVD, N (%)	563 (45)	421 (41)	142 (62)	< 0.001^P^
History of rheumatoid arthritis, N (%)	29 (2)	25 (2)	4 (2)	0.71^P^
History of disease in the GI tract, N (%)	183 (15)	153 (15)	30 (13)	0.58^P^
History of cancer, N (%)	72 (6)	53 (5)	19 (8)	0.087^P^
History of diabetes, N (%)	61 (5)	38 (4)	23 (10)	< 0.001^P^

For each variable, frequency is presented unless stated otherwise. The median (median absolute difference) is indicated with a symbol ‘*’. The *p* value (from either Mann–Whitney *U* test indicated with ‘M’ or Pearson's chi-squared test indicated with ‘P’) is for the difference in each variable between the survived and deceased participants.

*BMI* Body mass index, *CRP* C-reactive protein, *CVD* cardiovascular disease, *GI* gastrointestinal tract, *HDL* high-density lipoprotein, *IL* interleukin, *LDL* low-density lipoprotein, *TNF* tumor necrosis factor.

To explore the correlations and thus possible collinearities between cf-DNA and the other (continuous) health variables in the survival analysis, bivariate correlation analyses were performed and visualised as a heatmap. This analysis showed that cf-DNA correlates only modestly with the other health variables (all r ≤ 0.2) (see Supplementary Figure S1 online). The likelihood of issues arising due to collinearity between cf-DNA and the other variables in the multivariate Cox model was thus small.

The quality control analysis on the effect of storage time on cf-DNA levels indicated that while the absolute cf-DNA levels tended to increase with long storage, the rate of increase was constant across the samples (Supplementary Table S2). That is, the very high correlation (r ~ 0.97) between cf-DNA levels measured in the same samples ten years apart suggests that the storage-induced increase is proportional to the cf-DNA level so that the rank orders of the samples are maintained (Supplementary Table S2).

**Figure 1:**
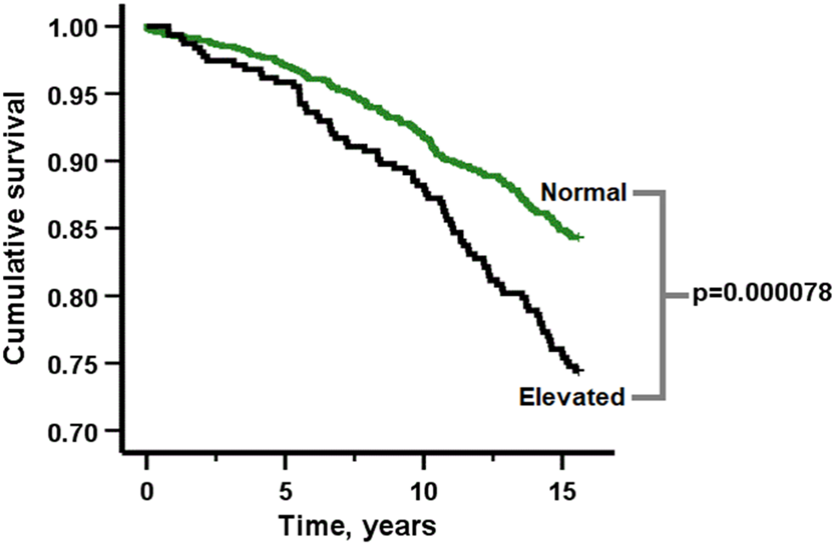
The estimated survival probabilities according to the baseline cf-DNA level divided into two groups, and pairwise comparison statistics between the groups. Individuals in the highest gender-wise cf-DNA quartile (n = 313, 80 [26%] deceased) were included to the group of “elevated cf-DNA levels” (black line) and all the other individuals (n = 944, 148 [16%] deceased) in the group of “cf-DNA level is in the normal range” (green line).

As shown in Fig. 1, mortality was higher in individuals with elevated cf-DNA levels than in those with cf-DNA levels in lower range. The relationship between the cf-DNA level tertiles and mortality was also dose-responsive, although the difference between the middle and highest tertile was only borderline significant (Supplementary Figure S2). In the Cox regression analysis, age and gender were significantly associated with mortality, also when adjusted for each other (Supplementary Table S3). Of the health indicators, cf-DNA, levels of fasting glucose, adiponectin, tumor necrosis factor (TNF)-alpha, interleukin (IL)-6, C-reactive protein (CRP), insulin, self-rated health (SRH), diabetes, CVD, respiratory disease, education level, vegetable consumption, smoking, alcohol consumption, and the frequency of intensive exercise were associated with mortality when adjusted for age and gender (*p* < 0.05, Supplementary Table S4). For cf-DNA, the hazard ratio (HR) for 0.1 µg/ml increase in cf-DNA was 1.022 (95% CI 1.013–1.030, *p* = 5 × 10^−7^, Supplementary Table S4), and the hazard ratio was very similar also at 5 years of follow-up (Supplementary Table S5). We chose to model the hazard for a 0.1 µg (i.e. 100 ng) increase in cf-DNA as it falls within the normal variation of cf-DNA; 0.1 µg/ml was the mean absolute difference in cf-DNA in our sample and thus represents a rather small, yet a biologically meaningful increase in the cf-DNA level.

In addition to age and gender, of the aforementioned health indicators that predicted mortality independent of age and gender, cf-DNA, SRH, smoking, fasting glucose and adiponectin remained significantly associated with mortality in the multivariate Cox analysis (*p* < 0.05), that is, these factors remained associated with mortality independently of each other. These predictors were hence kept in the final mortality risk model (Table 2). By considering a wider panel of markers in building of the final mortality model, instead of including only the standard mortality predictors, such as age, gender, smoking and plasma lipids, we were able to achieve a more accurate and critical assessment of the independent predictive value of cf-DNA. As some of the other markers (in Table 1) may contain the “same information” as cf-DNA with respect to mortality risk, exclusion of such markers could have led to overestimation of the predictive capability of cf-DNA.

**Table 2:** The final mortality risk model in the Health 2000 study population (all: n = 1,257), and in participants without (n = 694) and with (n = 563) the history of CVD.

	All			Without CVD			With CVD		
	HR	95% CI	*p*	HR	95% CI	*p*	HR	95% CI	*p*
Age, years	1.13	1.11–1.15	< 0.001	1.12	1.09–1.15	< 0.001	1.12	1.09–1.15	< 0.001
Gender, male	1.95	1.46–2.62	< 0.001	2.25	1.35–3.74	0.002	1.82	1.26–2.64	0.001
SRH	1.31	1.13–1.51	< 0.001	1.01	0.79–1.30	0.94	1.45	1.20–1.76	< 0.001
Smoking	2.65	1.95–3.59	< 0.001	2.38	1.43–3.96	< 0.001	2.90	1.97–4.28	< 0.001
Adiponectin, μg/ml	1.03	1.01–1.05	0.006	1.06	1.02–1.10	0.002	1.02	0.99–1.05	0.27
Fasting glucose, mmol/l	1.21	1.12–1.31	< 0.001	1.25	1.05–1.48	0.012	1.18	1.08–1.30	< 0.001
cf-DNA, 0.1 μg/ml	1.02	1.01–1.03	< 0.001	1.02	1.00–1.04	0.033	1.02	1.01–1.03	0.002

The mortality rates during the follow-up were 18% (n = 228), 12% (n = 86), and 25% (n = 142), respectively.

*CI* confidence interval, *cf-DNA* cell-free DNA, *HR* hazard ratio, *SRH* self-rated health.

Because the sample by design included a high proportion of participants with CVD (45%), the association between mortality and cf-DNA in the fully-adjusted final model was additionally analysed stratified by CVD status. The HRs of cf-DNA were similar across the strata; a 0.1 µg increase in cf-DNA was associated with a 2% increased risk of mortality in the full sample as well as in individuals with and without CVD (Table 2). Assumptions of proportional hazards were not violated by any of the predictors in the final Cox model.

Assessment of the predictive accuracy for the final model using Harrell’s C (Table 3, Model 9) demonstrated that the final model displayed a ‘good’ level of accuracy (Harrell’s C index: 0.80), yet individually none of the predictors performed at a good level. That is, cf-DNA and all the other variables, except age, performed alone at ‘poor’ or ‘weak’ level (Harrell’s C index < 0.7). To quantify the added value of cf-DNA to mortality prediction, the final Cox model without cf-DNA was compared to the final model with cf-DNA (Table 3: Models 8 → 9). The log-likelihood ratio test (LR-test) and Brier score indicated that the addition of cf-DNA improved model performance in terms of discrimination (Table 3).

**Table 3:** The mortality risk models (1–9) and goodness of fit statistics for the models in the Health 2000 survey (N = 1,257; follow-up 15 years).

	Model 1	Model 2	Model 3	Model 4	Model 5	Model 6	Model 7	Model 8	Model 9
**Age**									
HR	1.11	–	–	–	–	–	–	1.12	1.13
95% CI	1.10–1.13	–	–	–	–	–	–	1.10–1.14	1.11–1.15
*p*	4 × 10^−37^	–	–	–	–	–	–	3 × 10^−38^	5 × 10^−38^
**Gender, male**									
HR	–	2.06	–	–	–	–	–	2.18	1.95
95% CI	–	1.57–2.69	–	–	–	–	–	1.64–2.92	1.46–2.62
*p*	–	0.0000001	–	–	–	–	–	0.0000001	0.000008
**SRH**									
HR	–	–	1.60	–	–	–	–	1.33	1.31
95% CI	–	–	1.39–1.83	–	–	–	–	1.15–1.54	1.13–1.51
*p*	–	–	1 × 10^−11^	–	–	–	–	0.0001	0.0003
**Smoking**									
HR	–	–	–	1.99	–	–	–	2.76	2.65
95% CI	–	–	–	1.50–2.64	–	–	–	2.05–3.73	1.95–3.60
*p*	–	–	–	0.000002	–	–	–	3 × 10^−11^	4 × 10^−10^
**Adiponectin, μg/ml**									
HR	–	–	–	–	1.016	–	–	1.034	1.032
95% CI	–	–	–	–	0.994–1.038	–	–	1.012–1.058	1.009–1.055
*p*	–	–	–	–	0.15	–	–	0.003	0.006
**Fasting glucose, mmol/l**									
HR	–	–	–	–	–	1.21	–	1.21	1.21
95% CI	–	–	–	–	–	1.14–1.29	–	1.12–1.31	1.12–1.32
*p*	–	–	–	–	–	1 × 10^−9^	–	0.000002	0.000003
**cf**–**DNA, 0.1 μg/ml**									
HR	–	–	–	–	–	–	1.026	–	1.017
95% CI	–	–	–	–	–	–	1.018–1.033	–	1.008–1.026
*p*	–	–	–	–	–	–	1 × 10^−11^	–	0.0003
Harrell's C index	0.73	0.59	0.61	0.56	0.52	0.61	0.62	0.79	0.80
Log-likelihood (LR test p*)	–	–	–	–	–	–	–	− 1,460	− 1,454 (0.001)
Brier score	–	–	–	–	–	–	–	0.16	0.15

Hazard ratios (HRs) with 95% confidence intervals (CIs), and *p* values for the variables in the Cox regression analysis and Harrell’s C statistics are shown for each model. Metrics describing model improvement when adding cf-DNA to the risk model (model 8 → 9) are also shown. The variable units are according to Table 1.

*Model 9 is compared to model 8.

*CI* confidence interval, *HR* hazard ratio, *LR* likelihood ratio.

## Discussion

In this study, we analysed the association between cf-DNA and 15-year all-cause mortality and assessed whether cf-DNA adds value to mortality prediction on top of other risk factors. We built a multivariate mortality-prediction model from 30 health-related variables, including cf-DNA, with an a priori hypothesis that these variables are associated with mortality. These variables included blood biomarkers (levels of fasting glucose, adiponectin, TNF-alpha, IL-6, CRP, and insulin), education level, health behaviours (eating fresh vegetables, smoking, alcohol consumption, and frequency of intensive exercise), diseases (e.g. diabetes, cardiovascular diseases, respiratory diseases) and SRH. In addition to age and gender, of these factors, SRH, smoking, and levels of adiponectin, fasting glucose and cf-DNA remained independently associated with mortality and were considered as the final model predictors. The final mortality model showed good predictive accuracy as assessed by Harrell’s C. Comparing the discriminative ability of the final model with and without cf-DNA indicated that the addition of cf-DNA resulted in a rather small but significant increase in discrimination. The increased mortality risk conferred by elevated cf-DNA level was similar in magnitude in the full sample and in individuals with and without established CVD. The rather modest correlations between cf-DNA and the other mortality predictors imply that cf-DNA level likely represents a unique aspect in mortality risk, an aspect that is not covered by the other health indicators. Overall, these findings suggest that cf-DNA can be considered as a biomarker that is independent of other risk factors and sensitive enough to identify individuals at a higher mortality risk regardless of their previous positive history of CVD.

To date, only a limited number of studies have addressed the characteristics of cf-DNA as a mortality predictor in population-based cohorts. Previously, we reported that cf-DNA predicts 4-years all-cause mortality in very old individuals, independent of other risk factors^16^. In patient-based samples, cf-DNA predicts fatal outcomes in acute myocardial infarction, disease severity of pulmonary arterial hypertension^1,5,7–9^, and mortality in emergency department bacteraemia patients^3^. Our results thus add to the literature by showing that the predictive utility of cf-DNA is not limited to acute conditions or old individuals. Rather, it appears that cf-DNA is a broader marker of increased risk, also in middle-aged individuals and regardless of CVD status.

cf-DNA is an attractive biomarker for research purposes and for future use in clinical risk assessment, as quantification of this marker is a reasonably fast and cost-effective procedure. As done in this study, cf-DNA level can be determined using a simple fluorometric measurement with a DNA intercalating dye, in less than 30 min from the blood drawn. Thus, cf-DNA measurement could easily be implemented as a high-throughput technique; however, the current lack of standardisation of the methods hampers clinical translation. As there are several different ways to quantify cf-DNA, some of which require prior DNA extraction, more research is needed to determine the validity and reproducibility of each method. Our results indicate that cf-DNA levels alone may not be enough for accurate risk stratification for all-cause mortality in ostensibly healthy individuals, and thus, an optimal set of auxiliary variables should be identified. The predictive accuracy of cf-DNA level is likely to vary according to the outcome and sample characteristics; in a previous study of ours in bacteremia patients cf-DNA level alone showed a good predictive accuracy (area under the ROC curve 0.81) for case fatality^18^.

A number of studies have suggested that cf-DNA may have a role as an initiator or be a consequence of pathological processes, but the underlying mechanisms are not yet fully understood. For example, circulating cf-DNA seems to be involved in the pathophysiological changes of the endothelium: in trauma patients, degree of injury in endothelium and an increase in release of cf-DNA are linked together^19^. cf-DNA level is also elevated in the blood in association with increased endothelium damage during cardiac surgery, in an operation-time-dependent manner^15^. Increased levels of neutrophil extracellular traps (NETs) induce, in a dose-dependent manner, epithelial and endothelial cell death in the lung^20^. NETs is a pathogen-clearance-system utilised by neutrophils, and cf-DNA is an important compound in these traps. Thus, circulating cf-DNA might be an indicator of the magnitude of the damage as well as be involved in the damage-causing pathways.

The other predictors identified in the final survival model are in line with earlier observations. Higher age, male gender, smoking and poorer SRH are all common, established risk factors for mortality^21–24^. Higher levels of fasting glucose and adiponectin, both of which function in insulin signaling pathways, have likewise been associated with mortality in previous studies^25,26^. The fact that cf-DNA remained an independent mortality predictor when assessed in the same model with the aforementioned factors suggests that it explains a part of mortality not covered by the other markers. We have previously reported that cf-DNA associates with several inflammatory markers, suggesting that it reflects systemic inflammation^10,16,27^. Interestingly, the inflammatory markers analysed in this study—CRP, IL-6 and TNF-alpha—were no longer significant in the multivariate survival model. This finding would suggest that cf-DNA may be a stronger indicator of systemic inflammation pertinent to mortality. The finding that none of the disease diagnoses remained in the final model indicates that the blood biomarkers may capture the severity and subclinical forms of these diseases and thus perform better than clinically diagnosed diseases.

In summary, this is the first study showing that cf-DNA is independently associated with mortality in individuals aged 46–76 years. A comprehensive array of health information of the participants permitted us to assess the strength of cf-DNA as a mortality-predictor in comparison to other predictors. However, for generalisability and reproducibility, the results should be replicated in further populations with larger sample sizes. An obvious limitation of this study is its rather small sample size that did not allow us to analyse cause-specific mortality or stratify the analysis by specific age groups. In addition, storage of the plasma samples for 10 years before the cf-DNA assessment might have influenced the levels. However, our quality control analysis showed that despite the storage-induced increase in the absolute cf-DNA levels, the increase is proportional to the initial cf-DNA level so that the sample rank orders are maintained. The possible bias caused by long storage times to the relative cf-DNA values within a study is thus minimal. We nevertheless suggest that absolute cf-DNA levels in different studies are compared only when pre-analytical conditions and sampling procedures are identical.

The analyses herein focused on only the capacity of cf-DNA to predict all-cause mortality, and more research is needed to unravel the biological pathways that underlie the association between increased level of cf-DNA and mortality. Lastly, more analyses into the relationships between cf-DNA and the other mortality predictors representing different biological domains are needed.

In conclusion, our study identifies cf-DNA level as an independent predictor of all-cause mortality, both in individuals with and without established CVD. It also significantly improves discrimination when included to the model with the other risk factors. Our study thus strengthens the role of cf-DNA as a new viable marker of health and supports its further development towards clinical use.

## Methods

### Study population

The Health 2000 is a nationwide population-based survey (N = 8,028) in Finland that was performed in 2000–2001^17,28^. In 2001–2002, a subsample of the Health 2000 participants, aged 45–74 years at the baseline and living in six large cities (Helsinki, Turku, Tampere, Kuopio, Joensuu and Oulu) and their surroundings, was recruited for research focusing primarily on cardiovascular health (n = 1,526)^29^. Of the 1,526 participants, 1,257 individuals who had no missing data in any of the variables presented in Table 1 were included in the present study. As shown in Table 1, in this sample, 45% of the participants had CVD diagnosis at the baseline. More detailed information of the Health 2000 survey can be found at: https://thl.fi/en/web/thlfi-en/research-and-expertwork/projects-and-programmes/health-2000-2011.

### Variables in the analysis

For this study, we selected 30 health-related variables, including the cf-DNA level that we hypothesised to be associated with mortality (see statistical analysis). These variables are described in Table 1. These data were collected in health examinations, interviews and questionnaires in the surveys 2000–2001 and 2001–2002. The variables (lifestyle factors, education, and other diseases than diabetes and CVDs) were available only from the survey 2000–2001. All other data were collected in the survey 2001–2002. The information on different disease diagnoses (yes/no), smoking (yes/no), SRH, eating habits, and education level, originated from the interview, and the information on alcohol consumption and exercise originated from the questionnaire.

Education level corresponds to the total number of years in school, and this variable was categorised into tertiles. SRH was assessed with a question: “Is your present state of health: poor, rather poor, moderate, rather good or good?”. The body composition described by BMI (kg/m^2^) was based on measured height and weight. Fasting blood samples were collected in the health examination. A question, “During the past week, how often (number of days/week) have you eaten fresh vegetables (excluding potatoes)?” was used as an indicator of habitual vegetable consumption. As an indicator of alcohol consumption level, total quantity of alcohol (in grams) consumed in a week was used^30^. A question, “In a typical week during your leisure time, how often do you perform for more than 10 min such a physical activity that can be considered as an intense exercise (e.g. running, aerobic, heavy outdoor housekeeping)?” was used as an indicator of physical activity.

cf-DNA and other blood biomarkers were measured in EDTA plasma collected in the survey in 2001–2002. The plasma samples were centrifuged for 20 min at 1,800×*g* and stored at − 70 °C. cf-DNA level was quantified in 2012 from plasma that was thawed prior to analysis using a method described in Jylhava et al.^10^. Briefly, the level of cf-DNA in plasma was measured from the blood sample using a Quant-iT High-Sensitivity dsDNA Assay Kit and a Qubit Fluorometer v.1 (Invitrogen, Carlsbad, CA, USA) according to the manufacturer's instructions. The level of plasma ghrelin was measured according to Lähdeaho et al.^31^, and the level of plasma adiponectin according to Santaniemi et al.^32^. The other blood biomarkers, namely, levels of apolipoproteins A1 and B, fasting glucose, insulin, HDL, LDL and total cholesterol, triglycerides, resistin, CRP, IL-6, and TNF-alpha were analysed as described in Malo et al.^33^. Detailed assay dates are provided in the Supplementary Table S6.

The effect of storage time on cf-DNA levels was assessed by experimental quality control analysis. In specific, we now, in 2020, re-measured cf-DNA levels in 34 EDTA-plasma samples that were first measured right after collection in 2010 ^27^. These samples have been stored at − 70 °C throughout the time and thawed only once. Absolute median differences in the cf-DNA levels measured in 2010 vs. 2020 were assessed using the Mann–Whitney *U* test. Spearman rank correlations between the measurements in 2010 and 2020 were used to assess the degree to which the rank orders of the samples are maintained.

Indicator variables for having CVD or respiratory diseases were assigned so that in both cases having one or more disease diagnosis of either CVD or respiratory disease was coded as 1 and otherwise as 0. CVD diagnoses included myocardial infarction, coronary heart disease, heart failure, arrhythmia, hypertension, stroke, deep vein thrombosis, and other CVDs. Respiratory diseases included asthma, chronic obstructive pulmonary disease, chronic bronchitis, and other unspecified respiratory diseases. Indicator of diabetes diagnosis refers to any type of diabetes.

Dates of death were drawn on the 31st of December 2017 from the National Register on Causes of Death maintained by Statistics Finland. Mean length of the all-cause mortality follow-up was 15 (standard deviation 0.5) years.

### Statistical analysis

The difference in each study variable (in Table 1) between survivors and non-survivors was analysed using Mann–Whitney *U* test for continuous variables and Pearson's chi-squared test for categorical variables. Correlations, and thus potential collinearities in the survival model between cf-DNA and other continuous variables, were explored using Spearman's rank correlation coefficient statistics. The correlation matrix was ordered using hierarchical clustering and visualised as a heatmap using R-package ggcorrplot v0.1.3.

The relationship between cf-DNA and mortality was analysed and visualised using Kaplan–Meier cumulative survival curves. First, participants were categorised into two groups so that individuals in the highest gender-wise cf-DNA quartile are in the group of “elevated cf-DNA levels” and all other individuals in the group of “cf-DNA level is in the normal range” (Fig. 1). Then, to analyse whether cf-DNA level exhibits a dose-responsive relationship with mortality, it was categorised into tertiles (Supplementary Fig. 2). Differences between elevated and normal cf-DNA levels as well as across the cf-DNA tertiles were assessed using the log-rank test. In all other analyses, cf-DNA was treated as a continuous variable. For the subsequent Cox models (see below), cf-DNA values were multiplied by 100 so that the HR of cf-DNA would represent a risk associated with 0.1 μg/ml increase in the cf-DNA level.

Using Cox regression, we first analysed the univariate association of age and gender with mortality, and then adjusted the analysis of age with gender and vice versa. We then analysed individually all the variables in Table 1 for their associations with mortality, adjusting each model for age and gender. Those variables that remained significant were then entered simultaneously to a multivariate Cox model. Variables that remained significant (*p* < 0.05) in this multivariate Cox model were kept in the model, yielding a final mortality prediction model. Because cf-DNA has attracted attention in CVD medicine as a prognostic tool, and the sample by design includes a high proportion of participants with CVD, the association between mortality and cf-DNA in the fully-adjusted final model was also analysed stratified by CVD status. The proportional hazards assumption (i.e. independence of time) for the final Cox model and for each of the predictors in the model was evaluated using diagnostics based on the Schoenfeld residual correlation statistics. This was performed using cox.zph() function in the R-package survival v2.44-1.1.

Lastly, we analysed the added value of cf-DNA by using the following approaches suitable for censored data and nested models.

### Harrell’s C

We first assessed the predictive accuracies of all the final Cox model variables individually as well as for the full final model with and without cf-DNA. For this purpose, univariate Cox models were fit individually for all the final model predictors as well as for the full final model, with and without cf-DNA. After which the Harrell’s C statistics were calculated for each model using the cindex() function in the R-package dynpred. Harrell’s C is a concordance index that is appropriate for right-censored survival data as it assesses the amount of agreement (concordance) between predictions and outcomes by comparing the events and non-events, also accounting for events happened at different points in time.

### LR test

The LR test was used to assess whether the addition of cf-DNA to the final model improved model fit. The LR test was performed using the anova() function in R-package survival.

## Brier score

To quantify the magnitudes of the difference between predicted and observed events for the final model with and without cf-DNA, the Brier scores^34^ were calculated using the following formula.

$$\user2{ BS} = \frac{1}{{\varvec{n}}}\sum \left( {{\varvec{P}} - {\varvec{E}}} \right)^{2}$$

where n is the total number of individuals in the analysis. The probability of the event is estimated for each participant in the sample, separately for the model with and without cf-DNA. This calculation produces probability values *P*. Observed events (death, value 1) or survivals (value 0) at the end of the follow-up are assigned into a vector E. The Brier score takes values from 0 to 1 and assesses both discrimination and calibration, and estimates the calibration of these probabilities, that is, the level of confidence they provide. While there is no accepted range for a “good” Brier score, lower scores indicate better performance in terms of calibrated predictions.

The *p* value threshold was set to at value of 0.05. The data were processed, analysed and visualised using R studio, R version 3.6.1 (www.r-project.org) and the IBM SPSS software version 25.0 (IBM Corp., Armonk, New York, USA).

### Ethics declaration

Human participants were directly involved in the current study and only data was taken for the current study. The study was conducted in accordance with the Declaration of Helsinki ethical principles and all research participants gave their written informed consent to be part of the study. Recruitment of the subjects in the study was approved by the Ethical Committee for Epidemiology and Public Health at the Hospital District of Helsinki and Uusimaa^17^.

### Data sharing statement

Data used in the current study are not publicly available due to ethical reasons. However, data are available upon request from the Health 2000 survey for researchers who meet the criteria for access to confidential data. The Health 2000 data are available for researchers from the THL by request and based on study proposals approved by the scientific board of the survey (contact: terveys-2000–2011@thl.fi). The collaboration agreement concerning data transfer is required.

## Supplementary information

Supplementary file1
