# Supplementary material for: Circulating cell-free DNA level predicts all-cause mortality independent of other predictors in the Health 2000 survey
Source: Sci Rep. 2020 Aug 14;10:13809. doi: 10.1038/s41598-020-70526-9 (PMC7427793; doi:10.1038/s41598-020-70526-9)
Supplement: Supplementary file 1 — Supplementary file1 [file 41598_2020_70526_MOESM1_ESM.docx]

**Circulating cell-free DNA level predicts all-cause mortality independent of other predictors in the Health 2000 Survey**

L. Kananen^1,2^ *, M. Hurme^1,2^, M. Jylhä^2,3^, T. Härkänen^4^, S. Koskinen^4^, S. Stenholm^5,6^, M. Kähönen^7^, T. Lehtimäki^8^, O. Ukkola^9^, J. Jylhävä^2,10^ *

^1^ Faculty of Medicine and Health Technology (MET), Tampere University, Tampere, Finland

^2^ Gerontology Research Center (GEREC), Finland

^3^ Faculty of Social Sciences (Health Sciences), Tampere University, Tampere, Finland

^4^ Finnish Institute for Health and Welfare, Finland

^5^ Department of Public Health, University of Turku and Turku University Hospital, Turku, Finland

^6^ Centre for Population Health Research, University of Turku and Turku University Hospital; Turku, Finland

^7^ Faculty of Medicine and Health Technology, Tampere University, and Finnish Cardiovascular Research Center, Tampere, Finland; Department of Clinical Physiology, Tampere University Hospital, Tampere, Finland

^8^ Faculty of Medicine and Health Technology, Tampere University, and Finnish Cardiovascular Research Center, Tampere, Finland; Department of Clinical Chemistry, Fimlab Laboratories, Tampere, Finland

^9^ Research Unit of Internal Medicine, Medical Research Center Oulu, Oulu University Hospital, University of Oulu, Oulu, Finland

^10^ Department of Medical Epidemiology and Biostatistics, Karolinska Institutet, Stockholm, Sweden

Contact information:

L. Kananen laura.kananen@tuni.fi; *corresponding author

M. Hurme mikko.hurme@tuni.fi

M. Jylhä marja.jylha@tuni.fi

T. Härkänen tommi.harkanen@thl.fi

S. Stenholm sari.stenholm@utu.fi

S. Koskinen seppo.koskinen@thl.fi

M. Kähönen mika.kahonen@tuni.fi

T. Lehtimäki terho.lehtimaki@tuni.fi

O. Ukkola olavi.ukkola@oulu.fi

J. Jylhävä juulia.jylhava@ki.se; *corresponding author

**Supplementary material**

**Figures**


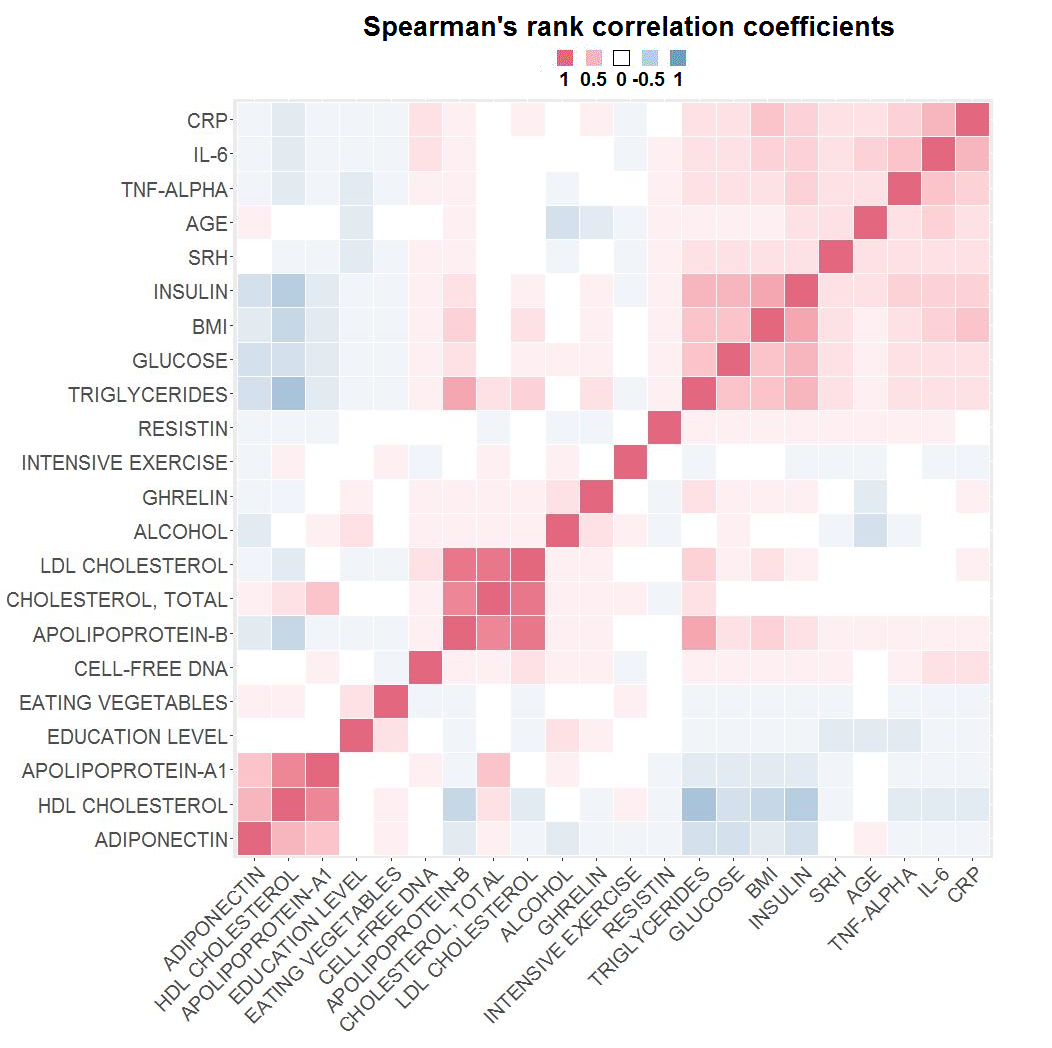


**Supplementary Figure S1.** Correlation analysis between cf-DNA level (residuals from the linear regression model adjusted for age and gender) and other continuous variables in the subsample of Health 2000 survey data (n=1,257) was performed using Spearman's rank correlation coefficient statistics. The correlation matrix was ordered using hierarchical clustering and visualised as a heatmap (p>0.05 with white color). Variable units are according to Table 1 and Supplementary Table S4.

**
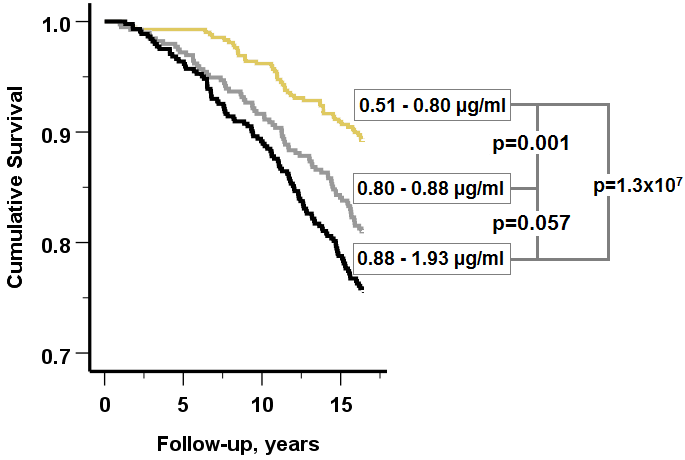
**

**Supplementary Figure S2.** The estimated survival probabilities according to the cf-DNA level divided into tertiles, and pairwise comparison statistics (log-rank test) across the cf-DNA level tertiles.

**Tables**

**Supplementary Table S1.** Primary causes of death in the sample according to ICD-10 codes.

|  | **ICD-10** | **Frequency** |
| --- | --- | --- |
| **Cancer** | C00-C97, B21 | 77 |
| **Non-stroke CVD** | I20-I25, I70, I73.9 | 41 |
| **Respiratory-related causes** | J00-J99 | 12 |
| **Stroke** | I60-I61, I63-I64 | 10 |
| **Other** | other codes | 88 |
|  | **Total** | 228 |
| Abbreviations: CVD=cardiovascular disease,  ICD-10=International Classification of Diseases, Tenth Revision | | |

**Supplementary Table S2.** Quality control analysis on the effect of ten years storage on cell-free DNA (cf-DNA) levels. The 34 samples that were first measured in 2010 (Jylhävä, J. et al. 2013) were now re-measured using the same analytical protocol and with two different Qubit Fluorometer versions (1 and 2) with version-specific assay reagents.

|  | **Spearman’s r (p-value)** | | |  | **Median**  **(interquartile range, IQR)** | |
| --- | --- | --- | --- | --- | --- | --- |
|  | **Qubit 1, in 2010** | **Qubit 1, in 2020** | **Qubit 2, in 2020** |  | **cf-DNA, µg/ml** | **p-value*** |
| **Qubit 1, in 2010** | 1.000 | 0.968 (8.6x10^-21^) | 0.966 (1.9x10^-20^) |  | 0.77 (0.22) |  |
| **Qubit 1, in 2020** | 0.968 (8.6x10^-21^) | 1.000 | 0.961 (4.8x10^-20^) |  | 1.17 (0.31) | 6.5x10^-11^ |
| **Qubit 2, in 2020** | 0.966 (1.9x10^-20^) | 0.961 (4.8x10^-20^) | 1.000 |  | 1.15 (0.34) | 5.8 x10^-11^ |
| *p-value for Mann-Whitney U test, cf-DNA levels measured in 2010 were compared to the levels measured in 2020, after ten years storage | | | | | | |

**Supplementary Table S3.** Associations of age and gender with mortality in the Health 2000 survey (n=1,257, follow-up 15 years) using Cox regression analysis. In model 1 age was analysed alone, in model 2 gender was analysed alone, and in model 3 age and gender were analysed together.

|  | **Variable** | **HR** | **95% CIs for HR** | | | **p** |
| --- | --- | --- | --- | --- | --- | --- |
| **Model 1** | Age | 1.12 | 1.10 | - | 1.13 | 4.0x10^-37^ |
| **Model 2** | Gender, male | 2.06 | 1.57 | - | 2.69 | 0.0000001 |
| **Model 3** | Age | 1.12 | 1.10 | - | 1.14 | 4.0x10^-38^ |
|  | Gender, male | 2.19 | 1.67 | - | 2.86 | 0.00000001 |
| Abbreviations: HR=Hazard ratio, CI=confidence interval | | | | | | |

**Supplementary Table S4.** Association between the health variables and all-cause mortality in the Health 2000 sample (n=1,257, follow-up 15 years) were analysed one by one using the Cox regression analysis adjusted for age and gender.

| **Domain** | **Variable** | **HR** | **95% CI for HR** | | | **p** | |  |  |
| --- | --- | --- | --- | --- | --- | --- | --- | --- | --- |
| **Lifestyle** | **Smoking status*** | **3.03** | **2.25** | **-** | **4.06** | | **1.7x10^-13^** | | |
| **Blood biomarker** | **Cell-free DNA, 0.1 ug/ml**** | **1.02** | **1.01** | **-** | **1.03** | | **0.0000005** | | |
| **Health status** | **Self-rated health, five categories**** | **1.44** | **1.24** | **-** | **1.66** | | **0.0000007** | | |
| **Blood biomarker** | **Fasting glucose, mmol/L**** | **1.20** | **1.11** | **-** | **1.29** | | **0.000004** | | |
| **Blood biomarker** | **IL-6, ng/L**** | **1.03** | **1.01** | **-** | **1.05** | | **0.0005** | | |
| **Blood biomarker** | **CRP, mg/L**** | **1.04** | **1.02** | **-** | **1.06** | | **0.0007** | | |
| **Disease** | **Diabetes**** | **1.98** | **1.29** | **-** | **3.05** | | **0.002** | | |
| **Disease** | **Any respiratory disease*** | **1.53** | **1.16** | **-** | **2.02** | | **0.003** | | |
| **Disease** | **Any cardiovascular disease**** | **1.51** | **1.15** | **-** | **1.99** | | **0.003** | | |
| **Blood biomarker** | **Fasting insulin, mU/L**** | **1.02** | **1.01** | **-** | **1.03** | | **0.005** | | |
| **Lifestyle** | **Intensive exercise**  **(>10min/day), days in a week*** | **0.92** | **0.86** | **-** | **0.99** | **0.018** | | |  |
| **Blood biomarker** | **TNF-alpha, ng/L**** | **1.02** | **1.00** | **-** | **1.04** | **0.020** | | |  |
| **Blood biomarker** | **Adiponectin, ug/ml**** | **1.03** | **1.00** | **-** | **1.05** | **0.022** | | |  |
| **Lifestyle** | **Eating fresh vegetables, days in a week*** | **0.86** | **0.75** | **-** | **0.98** | **0.022** | | |  |
| **Lifestyle** | **Alcohol consumption, g/week*** | **1.00** | **1.00** | **-** | **1.00** | **0.036** | | |  |
| **Socioeconomic** | **Education level, three categories*** | **0.84** | **0.70** | **-** | **0.99** | **0.038** | | |  |
| Blood biomarker | LDL cholesterol, mmol/L** | 0.87 | 0.75 | **-** | 1.01 | 0.067 | | |  |
| Blood biomarker | Total cholesterol, mmol/L** | 0.89 | 0.77 | **-** | 1.03 | 0.111 | | |  |
| Blood biomarker | Apolipoprotein B, g/L** | 0.64 | 0.37 | **-** | 1.12 | 0.118 | | |  |
| Blood biomarker | Ghrelin, pg/ml** | 1.00 | 1.00 | **-** | 1.00 | 0.141 | | |  |
| Disease | Rheumatoid arthritis* | 0.60 | 0.22 | **-** | 1.62 | 0.316 | | |  |
| Blood biomarker | Resistin, ng/ml** | 1.00 | 0.99 | **-** | 1.00 | 0.372 | | |  |
| Disease | Cancer* | 1.23 | 0.76 | **-** | 1.99 | 0.401 | | |  |
| Physiological  parameter | Body Mass Index, kg/m2** | 0.99 | 0.96 | **-** | 1.02 | 0.438 | | |  |
| Disease | Chronic disease in the gastrointestinal tract* | 0.90 | 0.61 | **-** | 1.32 | 0.576 | | |  |
| Blood biomarker | Apolipoprotein A1, g/L** | 0.91 | 0.56 | - | 1.46 | 0.683 | | |  |
| Blood biomarker | HDL cholesterol, mmol/L** | 1.05 | 0.75 | - | 1.46 | 0.775 | | |  |
| Blood biomarker | Triglycerides, mmol/L** | 1.02 | 0.88 | - | 1.18 | 0.818 | | |  |
| Abbreviations: BMI=Body mass index, CI=confidence interval, CRP=C-reactive protein, CVD=cardiovascular disease, GI=gastrointestinal tract, HDL=High-density lipoprotein, HR=Hazard ratio, IL=Interleukin, LDL=Low-density lipoprotein, TNF=Tumor necrosis factor. Significant predictors are shown in bold. Variables that were collected in the Health 2000 survey in 2000-2001 are indicated with *, and those collected in the survey 2001-2002 with **. The LDL cholesterol level was calculated with the Friedewald formula. | | | | | | | | |  |

**Supplementary Table S5.** Association between cf-DNA levels and all-cause mortality at different time-points in the Health 2000 sample (n=1,257) using the Cox regression analysis adjusted for age and gender. At 5-years-time-point 50 participants and at 15-years-time-point, 228 participants had died.

| **Model** | **HR** | **95% CI** | **p** |
| --- | --- | --- | --- |
| **5-years-follow-up** | 1.028 | 1.012 - 1.044 | 0.000527 |
| **15-years-follow-up** | 1.022 | 1.013 - 1.030 | 0.0000005 |
| Abbreviations: HR=Hazard ratio, CI=confidence interval | | | |

**Supplementary Table S6.** The laboratory assay dates of the blood biomarkers. The samples were stored at -70°C prior to the measurements.

| **Biomarker** | **Assay date (month/year)** |
| --- | --- |
| **Cell-free DNA, µg/ml** | 11/2012-12/2012 |
| **Adiponectin, µg/ml** | 2/2008-6/2008 |
| **Apolipoprotein A1, g/L** | 11/2003-4/2004 |
| **Apolipoprotein B, g/L** | 11/2003-4/2004 |
| **CRP, mg/L** | 10/2004-12/2004 |
| **Fasting glucose, mmol/L** | 11/2001-12/2002 |
| **Ghrelin, pg/ml** | 2/2008-6/2008 |
| **HDL cholesterol, mmol/L** | 11/2003-4/2004 |
| **IL-6, ng/L** | 10/2004-12/2004 |
| **Insulin, mU/L** | 11/2001-1/2003 |
| **LDL cholesterol, mmol/L** | 11/2003-4/2004 |
| **Resistin, ng/ml** | 2/2008-6/2008 |
| **TNF-alpha, ng/L** | 10/2004-12/2004 |
| **Total cholesterol, mmol/L** | 11/2003-4/2004 |
| **Triglycerides, mmol/L** | 11/2003-4/2004 |
| Abbreviations: CRP=C-reactive protein, CVD=cardiovascular disease, HDL=High-density lipoprotein, IL=Interleukin, LDL=Low-density lipoprotein, TNF=Tumor necrosis factor. | |

**References**

Jylhävä, J. et al. Characterization of the role of distinct plasma cell-free DNA species in age-associated inflammation and frailty. Aging Cell. 12, 388-397 (2013).
